# Parainfluenza virus 5 genomes are located in viral cytoplasmic bodies whilst the virus dismantles the interferon-induced antiviral state of cells

**DOI:** 10.1099/vir.0.012047-0

**Published:** 2009-09

**Authors:** T. S. Carlos, D. F. Young, M. Schneider, J. P. Simas, R. E. Randall

**Affiliations:** 1 School of Biology, University of St Andrews, Fife KY16 9ST, Scotland, UK; 2 Instituto de Microbiologia e Instituto de Medicina Molecular, Faculdade de Medicina, Universidade de Lisboa, Lisboa, Portugal

## Abstract

Although the replication cycle of parainfluenza virus type 5 (PIV5) is initially severely impaired in cells in an interferon (IFN)-induced antiviral state, the virus still targets STAT1 for degradation. As a consequence, the cells can no longer respond to IFN and after 24−48 h, they go out of the antiviral state and normal virus replication is established. Following infection of cells in an IFN-induced antiviral state, viral nucleocapsid proteins are initially localized within small cytoplasmic bodies, and appearance of these cytoplasmic bodies correlates with the loss of STAT1 from infected cells. *In situ* hybridization, using probes specific for the NP and L genes, demonstrated the presence of virus genomes within these cytoplasmic bodies. These viral cytoplasmic bodies do not co-localize with cellular markers for stress granules, cytoplasmic P-bodies or autophagosomes. Furthermore, they are not large insoluble aggregates of viral proteins and/or nucleocapsids, as they can simply and easily be dispersed by ‘cold-shocking’ live cells, a process that disrupts the cytoskeleton. Given that during *in vivo* infections, PIV5 will inevitably infect cells in an IFN-induced antiviral state, we suggest that these cytoplasmic bodies are areas in which PIV5 genomes reside whilst the virus dismantles the antiviral state of the cells. Consequently, viral cytoplasmic bodies may play an important part in the strategy that PIV5 uses to circumvent the IFN system.

## INTRODUCTION

The interferon (IFN) response is initiated when cells recognize that they have been infected by a virus and respond by producing IFN-*α*/*β*, which can act in a paracrine and autocrine manner to upregulate the expression of hundreds of cellular genes, the products of many having direct or indirect antiviral activity. Cells recognize that they have been infected by viruses by having specific intracellular and membrane-bound pattern-recognition receptors (PRRs) which recognize pathogen-associated molecular patterns (PAMPs) within certain products, such as double-stranded RNA or uncapped 5′ triphosphorylated single-stranded (ss)RNA, produced during virus replication and which are not found in uninfected cells. Two intracellular PRRs are the DExD/H-box RNA helicases, retinoic acid inducible gene I (RIG-I) and melanoma differentiation-associated gene 5 (mda-5). Once activated by their appropriate ligands, these PRRs initiate the IFN induction cascade which culminates in the production of IFN-*α*/*β* (for reviews see Pichlmair & Reis e Sousa, 2007; Saito & Gale, 2007; Randall & Goodbourn, 2008; Takeuchi & Akira, 2008, 2009). The secreted IFN-*α*/*β* then upregulates the expression of IFN-stimulated genes (ISGs) by activating the Jak/STAT pathway. Briefly, following binding of IFN-*α*/*β* to the type I IFN receptor, two kinases, Jak1 and tyk2, are activated and, as a result, phosphorylate the latent cytoplasmic transcription factors STAT1 and STAT2. These subsequently form stable heterodimers and migrate to the nucleus where, together with IFN regulatory factor (IRF)-9, they form an active transcription complex, termed ISGF3, that initiates transcription of the ISGs (reviewed by Stark *et al.*, 1998; Platanias, 2005; Randall & Goodbourn, 2008).

Although much has been learnt over recent years about how paramyxoviruses block aspects of the IFN response (Haller *et al.*, 2006; Fontana *et al.*, 2008; Randall & Goodbourn, 2008), much less is known about how viruses interact with cells in a pre-existing antiviral state, and yet this may be equally important to viral pathogenesis and epidemiology. Our studies on parainfluenza virus type 5 (PIV5; formerly known as SV5) have revealed that although the replication of PIV5 is severely affected in cells that are in an IFN-induced antiviral state prior to infection (Carlos *et al.*, 2005, 2007), the virus can dismantle the antiviral state and eventually replicate. It achieves this by targeting STAT1 for degradation and, since the cell cannot maintain the antiviral state indefinitely in the absence of continuous IFN signalling, a normal pattern of virus replication is established after 24–48 h (Precious *et al.*, 2007). When cells are in an IFN-induced antiviral state, as well as there being a dramatic change in the pattern of PIV5 protein synthesis (see below), there are also striking alterations in the distribution of the viral proteins within the infected cells (Carlos *et al.*, 2005, 2007). Most obviously, nucleocapsid proteins are located within viral cytoplasmic bodies rather than being more evenly distributed throughout the cytoplasm, as occurs in the absence of an IFN response (Carlos *et al.*, 2005). We have previously suggested that these viral cytoplasmic bodies are a defence mechanism in which the virus can hide from intracellular antiviral responses (Fearns *et al.*, 1994; Chatziandreou *et al.*, 2002; Carlos *et al.*, 2005). Here, we show that these viral cytoplasmic bodies are the first structures to be visualized when cells in an IFN-induced antiviral state become infected by PIV5. We also show that they contain viral genomes and that they are not insoluble aggregates as they can quickly be disrupted by cold-shocking live cells, a procedure that disrupts the cytoskeleton.

**Fig. 1. f1:**
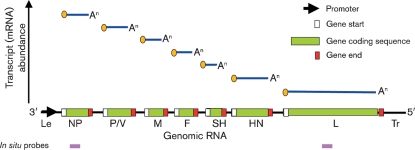
Schematic diagram of the gene order and the transcript abundance of PIV5 mRNAs (see text for details). The positions on the genome map that the NP and L *in situ* probes bind to are shown by pink boxes.

PIV5 is a member of the Paramyxoviridae, a family of enveloped viruses with single-stranded, negative-sense RNA genomes. The PIV5 genome is 15 246 nucleotides long and comprises seven genes (Fig. 1) encoding eight proteins, all of which are structural proteins. Like other paramyxoviruses, as well as transcribing individual mRNAs for the viral proteins, the virus must generate full-length antigenomic and genomic RNAs during replication. Both genomic and antigenomic RNAs are encapsidated by the nucleoprotein (NP), forming flexible helical nucleocapsids. Associated with the nucleocapsid is the viral polymerase complex comprising the viral large (L) and phospho (P) proteins. The V protein, which acts as an IFN antagonist by targeting STAT1 for proteasome-mediated degradation (Didcock *et al.*, 1999) and inhibiting the activity of mda-5 (Andrejeva *et al.*, 2004), is also found in low copy numbers within the virion. The nucleocapsid and associated proteins are surrounded by the virus envelope, which contains three integral membrane proteins – the haemagglutinin–neuraminidase (HN), fusion (F) and small hydrophobic (SH) proteins – the matrix (M) protein is located at the inner surface of the envelope. The viral polymerase initiates transcription by binding to a promoter at the 3′ end of genomic RNA and, by recognizing gene start and gene end sequences, sequentially synthesizes individual capped and polyadenylated mRNAs from the NP, V/P, M, F, SH, HN and L genes. Due to a specific RNA editing mechanism, the V/P gene encodes both the V and P proteins, with the V mRNA being a faithful transcript of the genomic RNA and the P mRNA having insertion of two non-templated G residues at the editing site (Thomas *et al.*, 1988). Since the polymerase only initiates transcription at the 3′ promoter but can disengage the further it proceeds along the genomic template, there is a transcriptional gradient in terms of the abundance of the individual mRNAs, with NP mRNA being the most abundant and L mRNA the least (Fig. 1; for reviews of the molecular biology of paramyxoviruses see Whelan *et al.*, 2004; Lamb & Parks, 2006).

Our previous studies have shown that following infection of cells in an IFN-induced antiviral state with PIV5, there are changes in the pattern of virus protein synthesis (Carlos *et al.*, 2005, 2007; Precious *et al.*, 2007). Whilst there is downregulation of all viral proteins synthesized, the expression levels of proteins downstream from the V/P gene are most severely affected. This can be partially explained by effects on transcription, in that there is an increase in the slope of the transcriptional gradient, possibly because in cells in an IFN-induced antiviral state the polymerase will disengage more readily from the genome during transcription. However, there is not complete concordance between the slope of the transcriptional gradient and the amount of virus protein present within infected cells; thus IFN must be inducing additional effects which affect the relative levels of the viral proteins (Carlos *et al.*, 2005).

## METHODS

### Cells, viruses and IFN.

Vero, MRC5 and A549 cells were grown as monolayers in 25 or 75 cm^3^ tissue culture flasks, in Dulbecco's modified Eagle's medium (DMEM) supplemented with 10 % (growth medium) or 2 % (maintenance medium) fetal calf serum (FCS) at 37 °C. When required, cells were treated with either Roferon A (Roche; human cells) or recombinant human IFN-*α*A/D [rHuIFN-*α*A/D; PBL Biomedical Laboratories; Vero cells] at 1000 units ml^−1^. PIV5 strains W3A (Choppin, 1964) and canine parainfluenza (CPI) (Baumgartner *et al.*, 1987) were grown and titrated under appropriate conditions in Vero cells.

### Plasmids.

Plasmids containing fragments of the NP and L genes of PIV5 were constructed by cloning PCR-amplified fragments with *Eco*RI and *Hin*dIII engineered sites for cloning into *Eco*RI–*Hin*dIII-digested pSPT19 plasmid (Roche), generating pSPT19/PIV5-NP (nt 452–842) and pSPT19/PIV5-L (nt 1953–2355). The plasmid pEH1.4 specifying the probe to mouse gammaherpesvirus-68 (MHV-68) tRNA 1–4 for use in *in situ* hybridization is as reported in Bowden *et al.* (1997).

### *In situ* hybridization and immunofluorescence.

Digoxigenin (DIG)-labelled ssRNA probes were generated by T7 or SP6 transcription of pSPT19/PIV5-NP or pSPT19/PIV5-L, using the DIG RNA Labelling kit (Roche) according to the manufacturer's instructions. SP6 RNA polymerase was used when preparing NP or L probes to detect RNA negative-sense viral genomes, while T7 RNA polymerase was used when preparing NP or L probes to detect positive-sense RNA antigenome or viral mRNAs. As a negative control, a DIG-labelled riboprobe encompassing MHV-68 vtRNAs 1–4 and microRNAs 1–6 was used, which was generated by T7 transcription of pEH1.4 (Bowden *et al.*, 1997). DIG-labelled riboprobes were subjected to LiCl/ethanol precipitation for removal of unincorporated DIG nucleotides. For *in situ* hybridization analysis, cells were grown on 13 mm diameter coverslips coated with poly-l-lysine, in individual wells of a six-well plate. Cells were pretreated for 14 h with 1000 units rHuIFN-*α*A/D ml^−1^, or left untreated followed by infection with either PIV5 W3A or CPI virus at high or low m.o.i. (or mock infection). After an adsorption period of 1–2 h, the virus inoculum (or maintenance medium for mock infections) was removed and replaced with fresh maintenance medium supplemented or not with rHuIFN-*α*. Depending on the experiment, the medium on the untreated cells was either supplemented with rHuIFN-*α* or left untreated as a control at 10 h post-infection (p.i.).

At various times p.i., monolayers were fixed (5 % formaldehyde and 2 % sucrose in PBS) for 15 min at room temperature, washed three times in PBS and then treated with 2 μg proteinase K ml^−1^ in 20 mM Tris pH 7.5/2 mM CaCl_2_ at 37 °C for 10 min. Proteinase K was removed and cell monolayers were washed twice with PBS containing 0.1 % Tween-20, refixed for 20 min at room temperature and permeabilized with 0.5 % Triton X-100 in PBS supplemented with 2 mM vanadyl ribonucleoside complex (Sigma) for 15 min at room temperature. Monolayers were subsequently washed three times with PBS and incubated with 2× sodium chloride–sodium citrate (SSC) with 0.025 % Tween-20 at 37 °C for 5 min. Cells were hybridized to DIG-labelled riboprobes in hybridization mix (Tris/HCl pH 8.0, NaH_2_PO_4_, Na_2_HPO_4_, Ficol, polyvinyl-pyrrolidone, SSC) supplemented with 50 % formamide, 0.5 mg sonicated salmon sperm ml^−1^, 0.5 mg tRNA ml^−1^, 1 mM DTT and RNase inhibitor (RNasin) at 55 °C overnight in a humidified chamber. Cells were washed for 15 min at room temperature with 2× SSC/10 mM Tris pH 7.5 and then washed with 0.1× SSC/10 mM Tris pH 7.5 for 15 min at room temperature. A stringent wash (30 % formamide, 0.1× SSC, 10 mM Tris pH 7.5) was carried out at 55 °C for 30 min. Cells were subsequently washed three times with TBST (150 mM NaCl, 10 mM KCl, 50 mM Tris pH 7.5, 0.1 % Tween-20), and then blocked at room temperature for 1 h with 2 % blocking reagent (Roche) in TBST supplemented with 20 % heat-inactivated sheep serum. Hybridized probe was detected by incubation of cell monolayers with alkaline-phosphatase-conjugated anti-DIG Fab fragments (Roche) diluted in TBST, 2 % blocking reagent, 1 % heat-inactivated sheep serum for 2 h at room temperature. When simultaneously detecting NP, the SV5-NP-a monoclonal antibody (Randall *et al.*, 1987) was appropriately diluted in the anti-DIG antibody solution. Cells were subsequently washed several times with TBST and the bound anti-DIG antibody conjugate was then visualized with the highly sensitive Fast Red Tablet (Roche) dissolved in 0.4 M NaCl/0.1 M Tris, pH 8.2 for 2 h at 37 °C. For simultaneous immunofluorescence, FITC-conjugated goat anti-mouse immunoglobulin was added to the Fast Red solution. The reaction was stopped by washing monolayers with 0.1 % Tween-20 in PBS, followed by several washes with PBS. For nuclear staining, cells were also stained with the DNA-binding fluorochrome DAPI (0.5 μg ml^−1^; Sigma-Aldrich) for 10 min at room temperature. Monolayers were washed in PBS and coverslips were mounted onto microscope slides in the presence of Mowiol mounting medium. A Leica DM5000B wide-field fluorescence microscope was used to examine cell monolayers.

### Immunofluorescence.

For immunofluorescence analysis that was carried out in the absence of *in situ* hybridization, cells were grown on 13 mm diameter coverslips (General Scientific) in individual wells of six-well or 24-well plates. Cells were infected with PIV5 CPI or W3A, and the inoculum was adsorbed for 1 h. Cells were treated with exogenous IFN at various times p.i. to monitor the virus replication cycle after virus transcription and replication had been established. At various times p.i., monolayers were incubated in fixing solution (5 % formaldehyde and 2 % sucrose in PBS) for 15 min at room temperature, then permeabilized (0.5 % Nonidet-P40 and 10 % sucrose in PBS) for 5 min, and washed three times in PBS containing 1 % FCS and 0.1 % azide (PBS, 1 % FCS, 0.1 % azide). To detect the proteins of interest, cell monolayers were incubated with 10–15 μl appropriately diluted primary antibody for 1 h. The antibody used to detect PIV5 NP was SV5-NP-a (Randall *et al.*, 1987), stress granules were detected using a rabbit polyclonal anti-Rck/p54 antibody (MBL International, code no. PD009) and STAT1 was detected with a rabbit polyclonal anti-STAT1 antibody (Abcam, ab 2071). Cells were subsequently washed (PBS, 1 % FCS, 0.1 % azide) several times and the antibody–antigen interactions were detected by indirect immunofluorescence (1 h incubation) with FITC- or Texas-red-conjugated goat anti-rabbit or anti-mouse immunoglobulin (Seralab), as appropriate. In addition, cells were stained with the DNA-binding fluorochrome DAPI (0.5 μg ml^−1^; Sigma-Aldrich) for nuclear staining. Following staining, monolayers were washed with PBS, mounted using either Citifluor AF-1 mounting solution (Citifluor) or Mowiol and examined using either a Nikon Microphot-FXA immunofluorescence microscope or a Zeiss LSM 5 Exciter confocal microscope.

## RESULTS

### Detection of virus genomes within CPI-infected Vero cells that have or have not been treated with IFN

**Fig. 2. f2:**
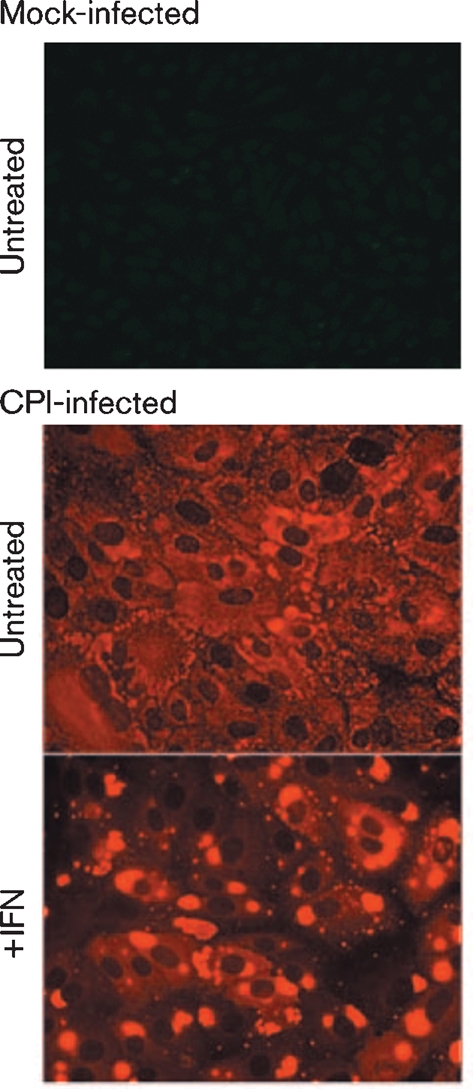
Visualization of viral cytoplasmic bodies in CPI-infected cells treated with IFN. Vero cell monolayers were infected with CPI at a high m.o.i. (50–100 p.f.u. per cell). After an adsorption period of 1–2 h on a rocking platform at 37 °C, the virus inoculum was removed and replaced with fresh maintenance medium. At 12 h p.i., the medium was either supplemented with rHuIFN-*α* or left untreated as a negative control. At 36 h post treatment, the cells were fixed and the distribution of the NP was visualized by immunofluorescence using a Nikon Microphot-FXA immunofluorescence microscope.

The CPI strain of PIV5 is unable to block IFN signalling due to three amino acid changes in its V protein that ablate its ability to target STAT1 for proteasome-mediated degradation (Chatziandreou *et al.*, 2002). However, CPI replicates normally in Vero cells (Carlos *et al.*, 2005, 2007; Precious *et al.*, 2007) since these cells cannot produce IFN (Desmyter *et al.*, 1968; Mosca & Pitha, 1986). Therefore, by infecting cells with CPI and adding IFN at various times p.i., it is possible to monitor the effects of IFN on the virus replication cycle after the virus transcription and replication has been established. In the absence of IFN, following infection of Vero cells with CPI, virus nucleocapsid proteins can be detected both throughout the cytoplasm and in viral cytoplasmic bodies. However, following addition of IFN to the culture medium of Vero cells which have been infected with CPI for 12 h (to allow virus replication to become established prior to the induction of an IFN-induced antiviral state), there is a marked alteration in the distribution of the viral nucleocapsid proteins compared with untreated cells, in that they are primarily visualized in cytoplasmic bodies at 36 h p.i. (Fig. 2 and Carlos *et al.*, 2005).

**Fig. 3. f3:**
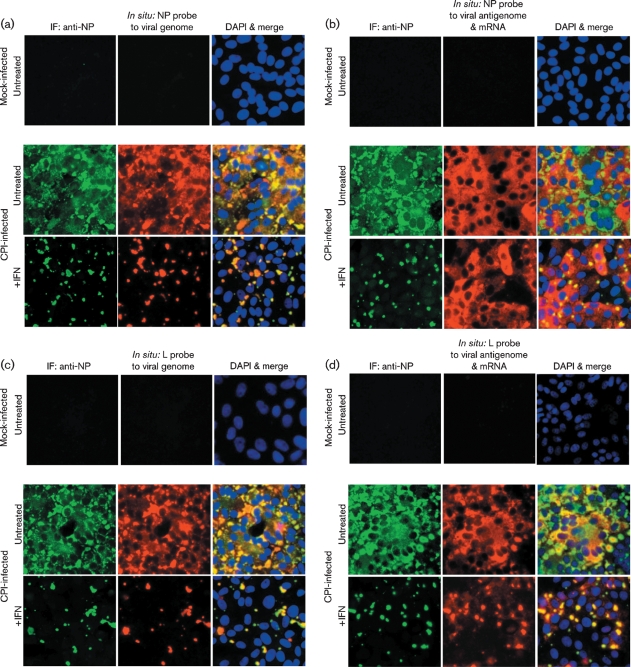
Detection of genomic RNA and antigenomic RNA/mRNA in CPI-infected cells. Vero cells were either mock-infected or infected with CPI at a high m.o.i., and at 8 h p.i., the cells were or were not treated with IFN. At 48 h p.i., the cells were fixed and co-stained by immunofluorescence, with an antibody to NP, and by *in situ* hybridization, using probes specific for genomic NP (a) or L (c) RNA or NP (b) or L (d) antigenomic RNA/mRNA. The cells were also counter-stained with DAPI to reveal the location of the nuclei. The final column in all four panels is the merged patterns from all three stains. Cells were visualized using a Leica DM5000B wide-field fluorescence microscope. Note: NP and L probes that bind to the genome should give the same intensity of staining, whilst the NP probe that binds to mRNA/antigenomes should give more intense staining than the L probe that binds to mRNA/antigenomes as the abundance of the NP mRNA is significantly greater than that of the L mRNA (see Fig. 1). IF, Immunofluorescence.

To determine whether virus genomes are present within these cytoplasmic bodies, we employed *in situ* hybridization using probes specific for sequences within the NP or L genes (Fig. 3a, c[Fig f3]), which are found at the 3′ and 5′ ends of the genome, respectively (Fig. 1). In addition, we also used probes that hybridized to viral NP or L mRNA, as well as to antigenomes (Fig. 3b, d[Fig f3]). It can be seen that, in general, the distribution of the virus genomes mirrored the distribution of the NP as visualized by immunofluorescence (Fig. 3a, c[Fig f3]). Thus, in the absence of IFN, virus genomes were detected throughout the cytoplasm as well as in cytoplasmic bodies, whilst after the cells had been treated with IFN, the virus genomes could only be detected in cytoplasmic bodies, which also stained with the anti-NP antibody. The pattern and intensity of the staining was similar regardless of whether a probe to genomic NP or L sequences was used (compare Fig. 3a, c[Fig f3]), consistent with the binding of the probes to genomic RNA. In contrast, there was little concordance between the distribution of the viral NP and the pattern of staining observed with the probes to viral mRNA and antigenomes (Fig. 3b, d[Fig f3]). Thus, in the absence of IFN, there was a more even distribution of the *in situ* probes throughout the cytoplasm, particularly the probe specific for NP sequences, presumably reflecting the distribution of viral mRNA. The intensity of the *in situ* hybridization was less when using the probe to L mRNA/antigenome sequences as compared with the NP probe (compare Fig. 3b, d[Fig f3]). This is consistent with the transcriptional gradient observed in paramyxovirus-infected cells, in which genes further away from the 3′ promoter are transcribed less frequently than those near the 3′ end of the genome. However, within this pattern of staining, cytoplasmic bodies could also be visualized, particularly with the L-specific probe. Furthermore, cytoplasmic bodies were more evident in cells treated with IFN, again particularly with the L-specific probe, which gave relatively little diffuse cytoplasmic staining. Presumably this reflects the abundance of NP mRNA compared with L mRNA and the relative abundance of mRNA to antigenomes in cells treated with IFN. Thus, these results suggest that in cells treated with IFN the diffuse staining reflects the distribution of viral mRNA and the viral antigenomes are primarily located with the cytoplasmic bodies.

Please note that the *in situ* RNA probes did not bind non-specifically to cytoplasmic bodies (Supplementary Fig. S1, available in JGV Online) and that viral cytoplasmic bodies are more resistant to the conditions used for *in situ* hybridization, i.e. proteinase K and heat treatment, than the NP, which is more diffusely distributed throughout the cytoplasm (Supplementary Fig. S2, available in JGV Online).

### Cells on the periphery of plaques developing in the presence of IFN often only contain small viral cytoplasmic bodies

**Fig. 4. f4:**
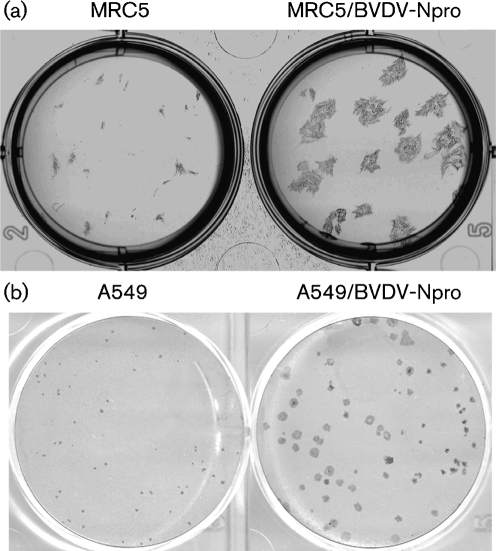
Plaques of PIV5 strain W3A formed on monolayers of MRC5, MRC5/BVDV-Npro, A549 and A549/BVDV-Npro. Note MRC/5BVDV-Npro and A549/BVDV-Npro cells cannot produce IFN in response to virus infection as BVDV-Npro targets IRF-3 for degradation (Hilton *et al.*, 2006). MRC5 cells were fixed at 6 days p.i., whilst the A549 cells were fixed at 10 days p.i.; both were immunostained with an antibody to PIV5 NP.

Unlike CPI, most PIV5 strains, including the W3A isolate, limit IFN production by interacting with mda-5 (Andrejeva *et al.*, 2004) and block IFN signalling by targeting STAT1 for proteasome-mediated degradation (Didcock *et al.*, 1999). Nevertheless, their ability to circumvent the IFN response is not absolute. This is illustrated by differences in the plaque size of PIV5 (W3A) in cells that can produce and respond to IFN compared with those in ‘IFN-compromised’ cells. For example, the plaque size of PIV5 (W3A) is significantly smaller in naïve MRC5 cells, which are non-transformed human fibroblasts that can produce and respond to IFN, compared with MRC5/BVDV-Npro cells, which cannot produce IFN because they constitutively express BVDV Npro, which targets IRF-3 for degradation (Hilton *et al.*, 2006) (Fig. 4a[Fig f4]). Similar results were seen following infection of A549 cells (human lung carcinoma cells that can also produce and respond to IFN) and A549/BVDV-Npro cells with PIV5 (W3A), although the relative size of the plaques was smaller than those observed using MRC5 cells (Fig. 4b[Fig f4]). Note that viruses, such as CPI, that cannot target STAT1 for degradation do not form plaques in cells that can produce and respond to IFN.

**Fig. 5. f5:**
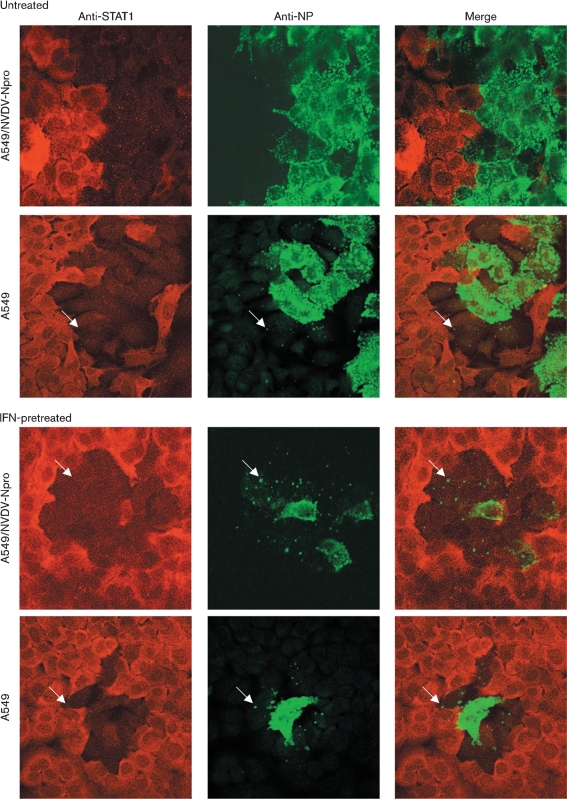
A549 and A549/BVDV-Npro cells were or were not pretreated with IFN for 18 h prior to infection with W3A at an m.o.i. of 0.01 p.f.u. per cell. At 4 days p.i., the cells were fixed and co-immunostained for STAT1 and PIV5 NP. Cells were visualized using a Zeiss LSM 5 Exciter confocal microscope. Arrows highlight cells at the edge of the plaque in which small viral cytoplasmic bodies can be detected and in which STAT1 has been degraded. A large, high-resolution copy of this image is available in JGV Online.

To better understand the dynamics of plaque formation in monolayers of cells that can produce and respond to IFN, we used immunofluorescence to monitor both viral NP synthesis and STAT1 degradation in A549 and A549/BVDV-Npro cells that were or were not pre-treated with IFN (Fig. 5). As expected from Fig. 4, plaques were significantly larger in A549/BVDV-Npro cells than in naïve A549 cells. Furthermore, in A549/BVDV-Npro cells, the cells at the edge of the plaque were generally strongly positive for NP and negative for STAT1. In contrast, in naïve A549, whilst the cells at the centre of the plaque were strongly positive for NP, NP was usually confined within small cytoplasmic bodies in cells at the edge of the developing plaque. However, strikingly, these cells were also negative for STAT1 staining. Presumably the presence of small viral cytoplasmic bodies in cells at the periphery of a developing plaque in A549 cells that had not been pre-treated with exogenous IFN reflects the fact that they had responded to endogenous IFN produced by cells already infected within the developing plaque, and were therefore in an IFN-induced antiviral state when they became infected. This conclusion is supported by the observation that pretreatment of either A549 or A549/BVDV-Npro cells (which can respond to IFN) with IFN reduced the plaque size further, but again, NP was normally only visualized in small viral cytoplasmic bodies in cells at the edge of the developing plaque and these cells were also negative for STAT1 (Fig. 5).

Note that, although STAT1 rapidly translocates to the nucleus upon treatment of cells with IFN, the nuclear localization of STAT1 is transient and by 2–4 h after treatment with IFN, the majority of STAT1 is located in the cytoplasm (Supplementary Fig. S3, available in JGV Online). Furthermore, expression of STAT1 is upregulated by IFN and as a consequence, the majority of STAT1 is unphosphorylated and remains in the cytoplasm. Together, these observations explain the primarily cytoplasmic distribution of STAT1 observed in cells exposed to IFN in Fig. 5.

**Fig. 6. f6:**
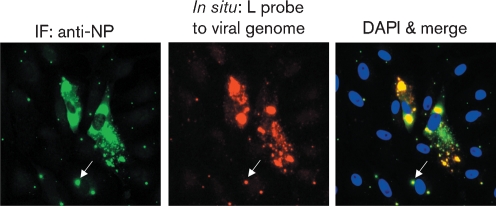
Detection of viral genomes in PIV5 cytoplasmic bodies in cells on the periphery of a developing plaque as PIV5 (W3A) spreads through a monolayer of cells in an IFN-induced antiviral state. Vero cells were infected with W3A at an m.o.i. of 0.01 p.f.u. per cell. At 8 h p.i., IFN was added to the culture medium and, at 48 h p.i., the cells were fixed; the presence of NP was detected by immunofluorescence and genomic RNA was detected using a probe specific for L gene sequences as described in the legend to Fig. 2. Note the presence of low numbers of small viral cytoplasmic bodies (an example of which is indicated with arrows) in the cells surrounding the two cells in which large amounts of viral NP proteins and genomic RNA can be detected. Cells were visualized using a Leica DM5000B wide-field fluorescence microscope. IF, Immunofluorescence.

Similar results were obtained with Vero cells, which cannot produce IFN. In the absence of IFN, plaques on Vero cells were large and the cells at the edge of a developing plaque were generally strongly positive for NP and negative for STAT1. In contrast, in monolayers treated with IFN, the plaques were much smaller and in many of the cells at the periphery of developing plaques, the NP protein could only be visualized in a few, small cytoplasmic bodies. Again, strikingly, STAT1 was degraded even in cells in which NP could only be detected in viral cytoplasmic bodies (Supplementary Fig. S4, available in JGV Online). Evidence for the presence of genomic RNA within these small cytoplasmic bodies, visualized around developing plaques, was obtained by *in situ* hybridization. Vero cells were infected with W3A at a low m.o.i. and, at 8 h p.i., IFN was added to the culture medium. At 48 h p.i., the cells were fixed and viral genomic RNA was detected using a probe specific for genomic L sequences. In Fig. 6, two cells are visible in which large amounts of virus antigen and genomic RNA can be detected that presumably represent the initially infected cells. However, in the surrounding cells, small cytoplasmic bodies which are positive for both NP and genomic RNA are clearly visible.

### PIV5 cytoplasmic bodies do not co-localize with autophagosomes, P-bodies or stress granules and are not insoluble aggregates of proteins and RNA

**Fig. 7. f7:**
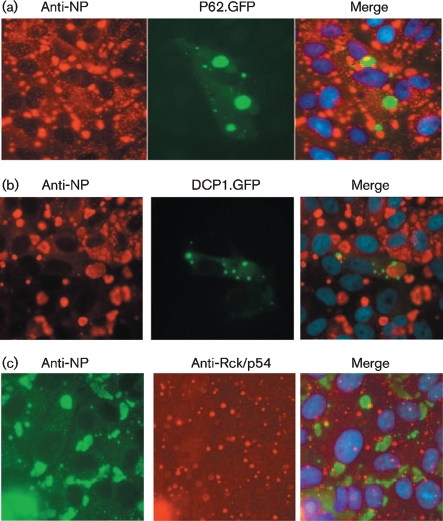
Viral cytoplasmic bodies do not co-localize with autophagosomes, cellular cytoplasmic P-bodies or stress granules. Vero cells were transfected with plasmids expressing GFP-tagged marker proteins for autophagosomes (a; P62.GFP) or P-bodies (b; DCP1.GFP). At 24 h post-transfection, the cells were infected with CPI and 12 h later, they were treated with IFN. At 18 h post-treatment, the cells were fixed and immunostained for NP. (c) Untransfected cells were infected and treated with IFN as above and co-stained with an anti-Rck/p54 antibody and an antibody to PIV5 NP. Cells were visualized using a Nikon Microphot-FXA immunofluorescence microscope. No co-localization of the marker proteins and viral proteins was observed.

Morphologically, PIV5 cytoplasmic bodies loosely resemble cellular structures, such as autophagosomes, P-bodies or stress granules. Autophagy is a process that leads to the degradation of long-lived proteins in the cell but it also has a role in innate defences against virus infections (reviewed by Lee & Iwasaki, 2008). mRNA stalled in translation or targeted for degradation may be located in stress granules and/or P-bodies. Furthermore, it has been reported that some viral RNAs and proteins, as well as host cell proteins with antiviral activity, accumulate in stress granules/P-bodies (reviewed by Beckham & Parker, 2008). To determine whether PIV5 cytoplasmic bodies were associated with autophagosomes or P-bodies, Vero cells were transfected with plasmids P62.GFP (Bjorkoy *et al.*, 2005) and DCP1.GFP (van Dijk *et al.*, 2002) expressing GFP-tagged marker proteins for autophagosomes or P-bodies, respectively. At 24 h post-transfection, the cells were infected with CPI and were treated with IFN 12 h later. At 18 h post-treatment, the cells were fixed and the distribution of the markers was compared with that of the viral cytoplasmic bodies. No co-localization of either marker was observed with PIV5 cytoplasmic bodies (Fig. 7a, b[Fig f7]). Furthermore, using immunofluorescence, no co-localization between PIV5 cytoplasmic bodies and Rck/p54, a marker for cellular stress granules, was observed (Fig. 7c[Fig f7]). Indeed, using antibodies against a variety of markers to cellular structures, including peroxisomes, endosomes, COP II vesicles, Golgi compartments and actin, we have so far failed to visualize the co-localization of any cellular protein/structure with PIV5 cytoplasmic bodies (data not shown).

**Fig. 8. f8:**
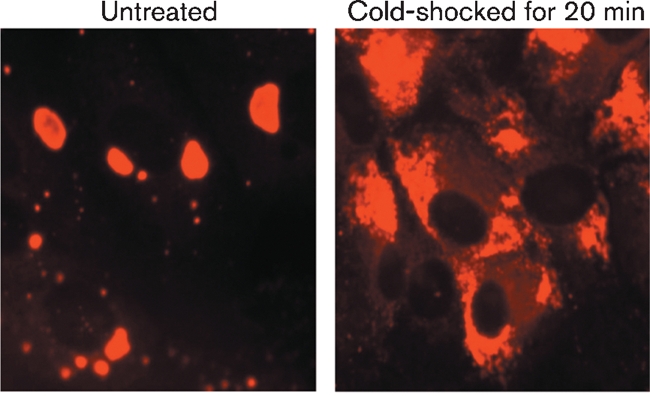
Viral cytoplasmic bodies are not large insoluble aggregates of viral proteins. Vero cells were infected with PIV5 CPI at a high m.o.i. and were treated with IFN at 8 h p.i. At 48 h p.i., the cells were or were not cold-shocked for 20 min with ice-cold PBS, after which time the cells were fixed and the distribution of the PIV5 NP visualized by immunofluorescence using a Nikon Microphot-FXA immunofluorescence microscope.

Although PIV5 cytoplasmic bodies did not co-localize with autophagosomes, it was possible that they were insoluble large aggregates of viral proteins and nucleocapsids. However, whilst manipulating live cells, we noted, as shown in Fig. 8, that it was relatively easy to disrupt PIV5 cytoplasmic bodies by treating the cells with cold PBS (a process known to disrupt microtubules, reviewed by Al-Fageeh & Smales, 2006) prior to fixation for immunofluorescence analysis (Fig. 8), thereby demonstrating that they are not insoluble aggregates of protein and/or nucleocapsids.

## DISCUSSION

Although PIV5 limits the amount of IFN induced, and targets STAT1 for proteasome-mediated degradation, its ability to circumvent the IFN response is not absolute, as witnessed by the fact that PIV5 forms smaller plaques on ‘IFN-competent’ cells compared with ‘IFN-compromised’ cells (Fig. 4, see also Young *et al.*, 2003). *In vivo* cells, such as plasmacytoid dendritic cells (pDCs), will also produce large amounts of IFN in the context of an overall immune response to virus infection (Liu, 2005). Consequently, at some point during an on-going *in vivo* infection, PIV5 will undoubtedly encounter and infect cells that are already in an IFN-induced antiviral state and the way in which PIV5 (and other viruses) interacts with such cells is likely to influence its pathogenesis and epidemiology. Results presented here, and elsewhere, provide a model for how PIV5 can dismantle an IFN-induced antiviral state of cells, and suggest a mechanism by which PIV5 is able to establish prolonged or even persistent infections *in vivo*. Our working model is that upon infection of cells in an IFN-induced antiviral state, although virus transcription and protein synthesis are initially severely affected (Carlos *et al.*, 2005, 2007), PIV5 targets STAT1 for proteasome-mediated degradation. With the resulting cessation of IFN signalling, the cells eventually exit their antiviral state and normal virus replication is established (Precious *et al.*, 2007). However, until the cells leave the antiviral state, the virus has to maintain its genome in a functional state within the infected cell. *In situ* hybridization evidence presented here shows that viral genomes are primarily located in cytoplasmic bodies in cells in an IFN-induced antiviral state. It is only when the cells exit their antiviral state and normal virus replication is established that a more diffuse cytoplasmic distribution of the nucleocapsid proteins and genomic RNA is observed.

Whilst the biochemical nature of PIV5 cytoplasmic bodies has to be fully investigated, a number of conclusions can be drawn. Firstly, whilst PIV5 cytoplasmic bodies clearly contain genomic RNA, they also probably contain antigenomic RNA. This latter conclusion is based upon the observation that *in situ* hybridization using the L probe to antigenomic RNA/mRNA readily stains the cytoplasmic bodies and gives only weak, diffuse cytoplasmic staining, whilst staining with the NP probe to antigenomic RNA/mRNA gives much stronger diffuse cytoplasmic staining (although the cytoplasmic bodies can also be observed). The diffuse cytoplasmic staining is therefore likely to represent mRNA, whilst the cytoplasmic body staining is likely to be due to the presence of antigenomic RNA. Secondly, PIV5 cytoplasmic bodies are not large insoluble aggregates of viral nucleocapsids or nucleocapsid proteins as they can quickly and easily be disrupted by cold-shocking cells with PBS, a process known to disrupt microtubules (Al-Fageeh & Smales, 2006). Thirdly, the cytoplasmic bodies do not co-localize with markers of autophagosomes, P-bodies or stress granules, and indeed, we have so far failed to detect any cellular protein that localizes within or around the viral cytoplasmic bodies, suggesting they are areas from which cellular proteins may be excluded. Fourthly, since the L protein can also be detected in these cytoplasmic bodies (data not shown), active transcription and replication may occur within the cytoplasmic bodies, and this is something we are currently investigating.

The natural history of PIV5 is poorly understood. It has been isolated on multiple occasions from a variety of species, and has been linked to prolonged/persistent infections. For example, it has regularly been isolated from human tissues and bone marrow cultures, where presumably it must have established a persistent infection. Furthermore, PIV5 is also regularly isolated in diagnostic laboratories, although often the original source of virus is unclear and a matter of debate (reviewed and discussed in Chatziandreou *et al.*, 2004). Thus, PIV5 is a virus that continues to circulate within the environment, usually without having an obvious effect on human or animal health. Thus, it is possible that the way in which PIV5 interacts with the IFN system, including the formation of cytoplasmic bodies during its relatively slow spread from cell to cell in the presence of IFN, may lead to a prolonged, low-grade infection. This in turn may lead to a situation in which infected hosts may not be particularly infectious (or ill) but who would shed virus over prolonged periods of time, thereby influencing virus epidemiology.

## Supplementary Material

[Supplementary Material]
