# Supplementary material for: Parainfluenza virus 5 genomes are located in viral cytoplasmic bodies whilst the virus dismantles the interferon-induced antiviral state of cells
Source: J Gen Virol. 2009 Sep;90(Pt 9):2147–56. doi: 10.1099/vir.0.012047-0 (PMC2885057; doi:10.1099/vir.0.012047-0)
Supplement: [Supplementary Material] [file supp_90_9_2147__index.html]

 Parainfluenza virus 5 genomes are located in viral cytoplasmic bodies whilst the virus dismantles the interferon-induced antiviral state of cells -- Carlos et al. 90 (9): 2147 Data Supplement - Supplementary Material -- Journal of General Virology

### Parainfluenza virus 5 genomes are located in viral cytoplasmic bodies whilst the virus dismantles the interferon-induced antiviral state of cells, by T. S. Carlos, D. F. Young, M. Schneider, J. P. Simas and R. E. Randall

*Journal of General Virology* vol. **90**, part 9, pp. 2147 - 2156

**Supplementary Fig. S1.** PIV5 cytoplasmic bodies do not bind RNA probes non-specifically.

**Supplementary Fig. S2.** When counter-immunostaining cells which had been subjected to *in situ* hybridization, the diffuse pattern of cytoplasmic staining with anti-NP antibodies was less intense and the PIV5 cytoplasmic bodies were more evident than we had previously observed.

**Supplementary Fig. S3.** IFN-induced translocation of STAT1 to the nucleus is transient.

**Supplementary Fig. S4.** Vero cells were infected with PIV5 (W3A), with or without IFN added, which highlighted a cell at the edge of a plaque in which small viral cytoplasmic bodies were detected and in which STAT1 was degraded.

[Single PDF of Figures] (258 KB)

**Fig. 5 of the main paper.** A549 and A549/BVDV–Npro cells were or were not pretreated with IFN for 18 h prior to infection with W3A. A high-resolution version of this figure is available here (4 MB).

  
  
