# Supplementary material for: Parainfluenza virus 5 genomes are located in viral cytoplasmic bodies whilst the virus dismantles the interferon-induced antiviral state of cells
Source: J Gen Virol. 2009 Sep;90(Pt 9):2147–56. doi: 10.1099/vir.0.012047-0 (PMC2885057; doi:10.1099/vir.0.012047-0)
Supplement: [Supplementary Material] [file supp_90_9_2147__1.pdf]

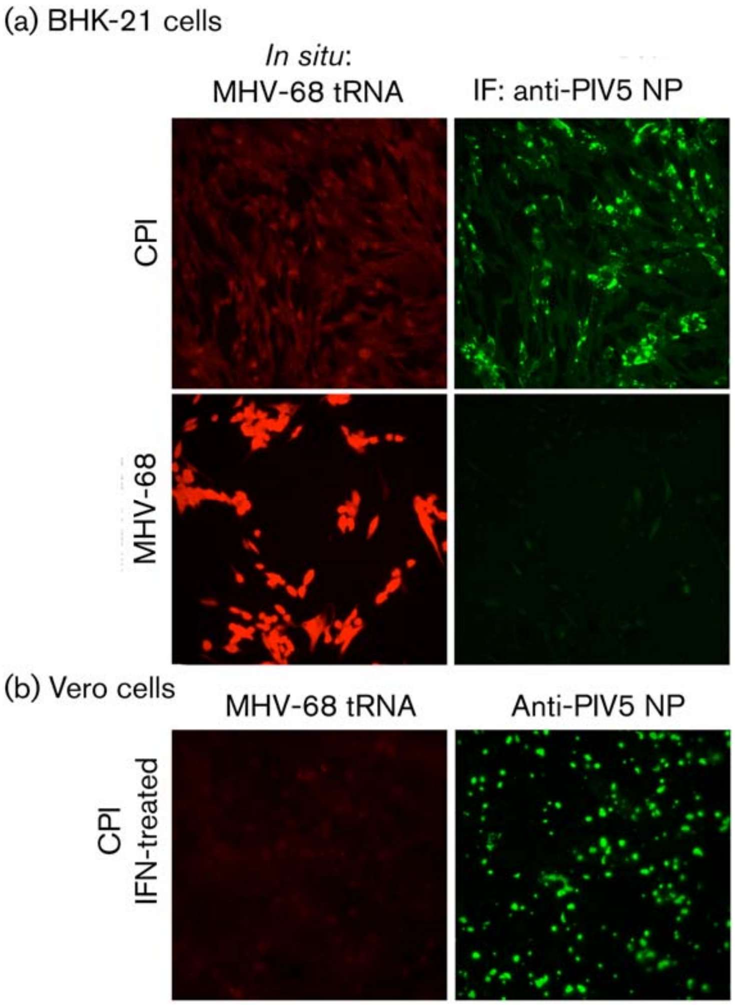

Supplementary Fig. S1. PIV5 cytoplasmic bodies do not bind RNA probes non-specifically. BHK and Vero cells were infected with either MHV-68 or CPI at high m.o.i. (10 p.f.u. per cell) and were or were not treated with IFN at 8 h p.i. At 48 h p.i., the cells were fixed and co-stained by immunofluorescence, with an antibody to PIV5 NP, and by *in situ* hybridization, using a probe specific for MHV-68 tRNAs 1–4. Cells were visualized using a Leica DM5000B wide-field fluorescence microscope. Note: the reason for using BHK cells is that MHV-68 does not productively infect Vero cells.

Carlos, T. S., Young, D. F., Schneider, M., Simas, J. P. and Randall, R. E. (2009). Parainfluenza virus 5 genomes are located in viral cytoplasmic bodies whilst the virus dismantles the interferon-induced antiviral state of cells. *J Gen Virol* **90**, 2147–2156.

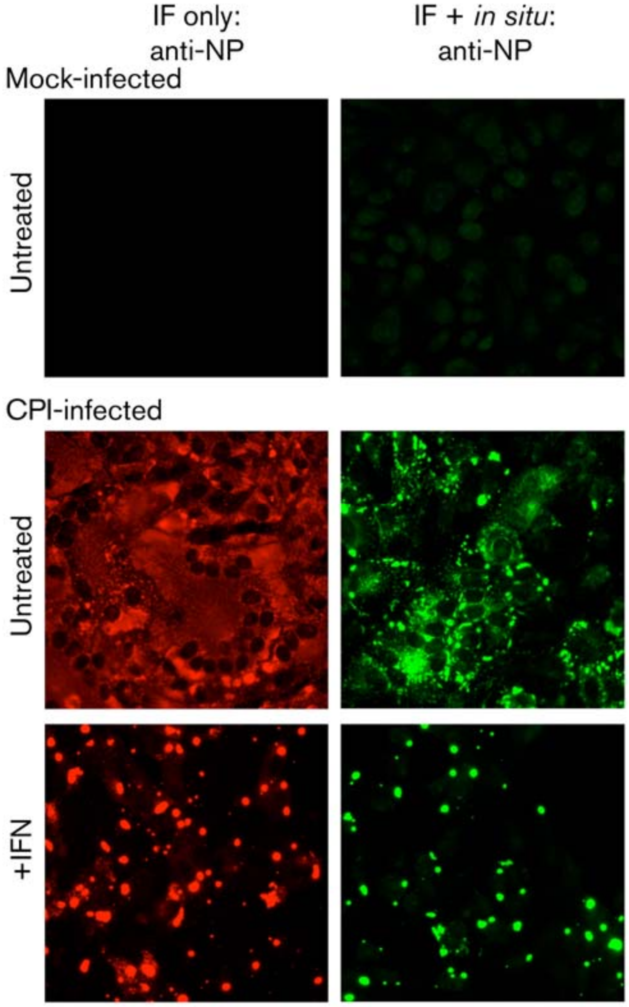

Supplementary Fig. S2. When counter-immunostaining cells which had been subjected to *in situ* hybridization, the diffuse pattern of cytoplasmic staining with anti-NP antibodies was less intense and the PIV5 cytoplasmic bodies were more evident than we had previously observed, i.e. when the cells were fixed and permeabilized just for immunofluorescence (compare Figs 2 and 3 of the main paper). This was because the viral cytoplasmic bodies were more resistant to the conditions used for *in situ* hybridization, proteinase K and heat treatment, than the NP that is more diffusely distributed throughout the cytoplasm. Vero cells were either mock-infected or infected with CPI, at a high m.o.i., and were or were not treated with IFN 8 h p.i. At 48 h p.i., the cells were fixed and treated either using our 'usual' conditions for immunofluorescence (i.e. formaldehyde fixation followed by permeabilization) or as they would be for *in situ* hybridization (see text of the main paper for details) prior to being stained for immunofluorescence with an antibody to the NP. Cells were visualized using a Leica DM5000B wide-field fluorescence microscope.

Carlos, T. S., Young, D. F., Schneider, M., Simas, J. P. and Randall, R. E. (2009). Parainfluenza virus 5 genomes are located in viral cytoplasmic bodies whilst the virus dismantles the interferon-induced antiviral state of cells. *J Gen Virol* **90**, 2147–2156.

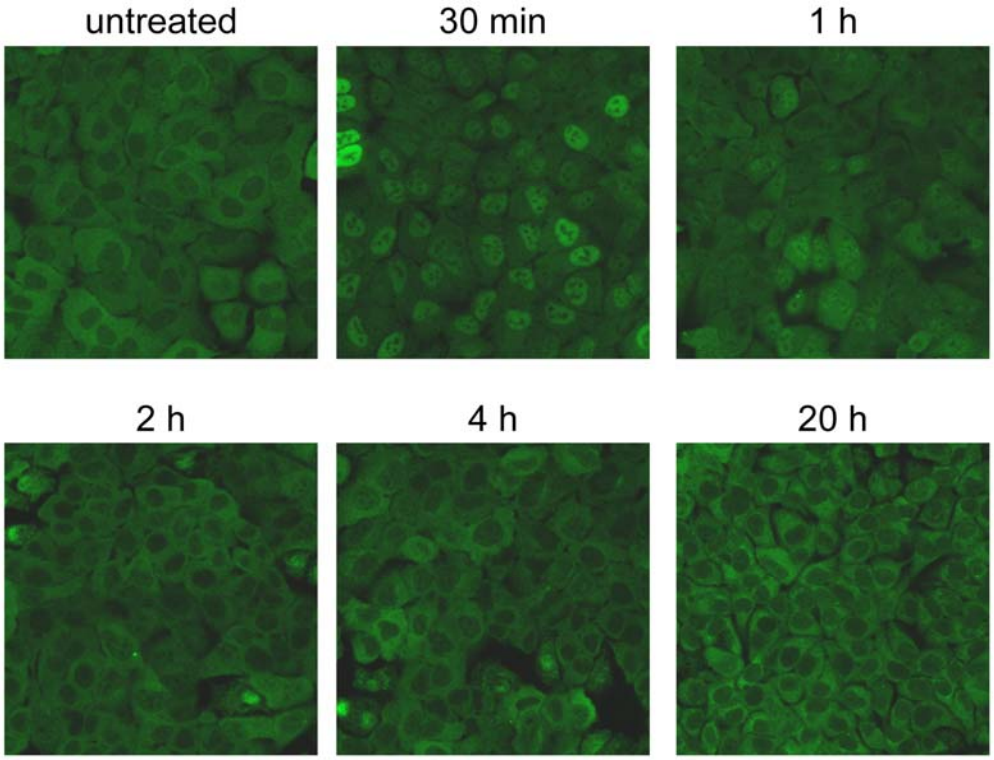

Supplementary Fig. S3. IFN-induced translocation of STAT1 to the nucleus is transient. A549 cells were or were not treated with IFN and were immunostained for STAT1 at various times post treatment. Note that following IFN-treatment, STAT1 rapidly translocates to the nucleus (30 min), but after 1 h treatment with IFN, STAT1 is primarily localized in the cytoplasm.

Carlos, T. S., Young, D. F., Schneider, M., Simas, J. P. and Randall, R. E. (2009). Parainfluenza virus 5 genomes are located in viral cytoplasmic bodies whilst the virus dismantles the interferon-induced antiviral state of cells. *J Gen Virol* **90**, 2147–2156.

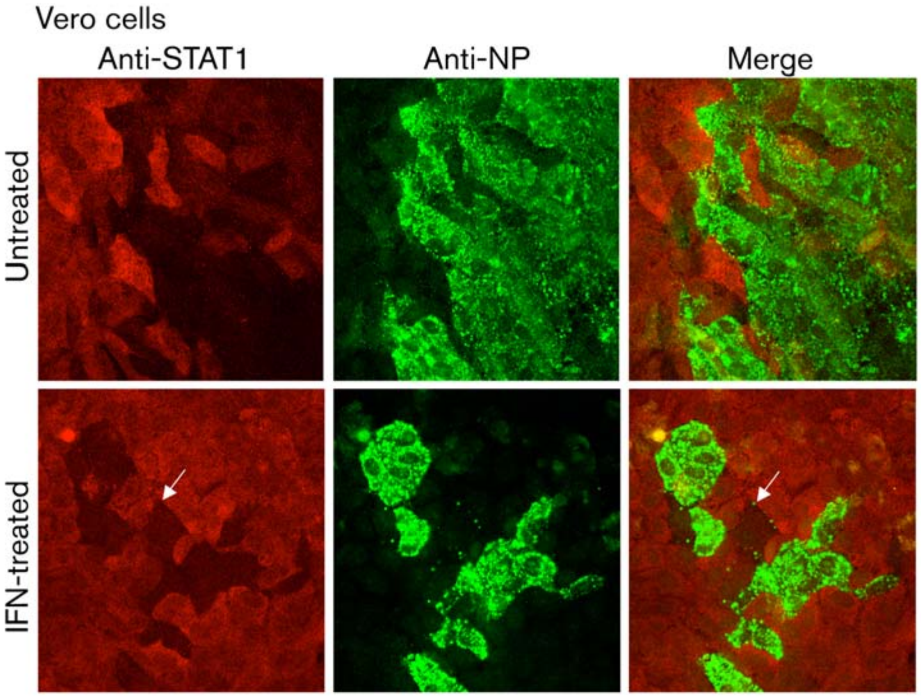

Supplementary Fig. S4. Vero cells were infected with PIV5 (W3A) at an m.o.i. of 0.01 p.f.u. per cell and IFN was or was not added to the culture medium at 8 h p.i. At 4 days p.i., the cells were fixed and coimmunostained for STAT1 and PIV5 NP. Cells were visualized using a Zeiss LSM 5 Exciter confocal microscope. Arrows highlight a cell at the edge of plaque in which small viral cytoplasmic bodies can be detected and in which STAT1 has been degraded.
