# Supplementary figures and images for: Parainfluenza virus 5 genomes are located in viral cytoplasmic bodies whilst the virus dismantles the interferon-induced antiviral state of cells
Source: J Gen Virol. 2009 Sep;90(Pt 9):2147–56. doi: 10.1099/vir.0.012047-0 (PMC2885057; doi:10.1099/vir.0.012047-0)

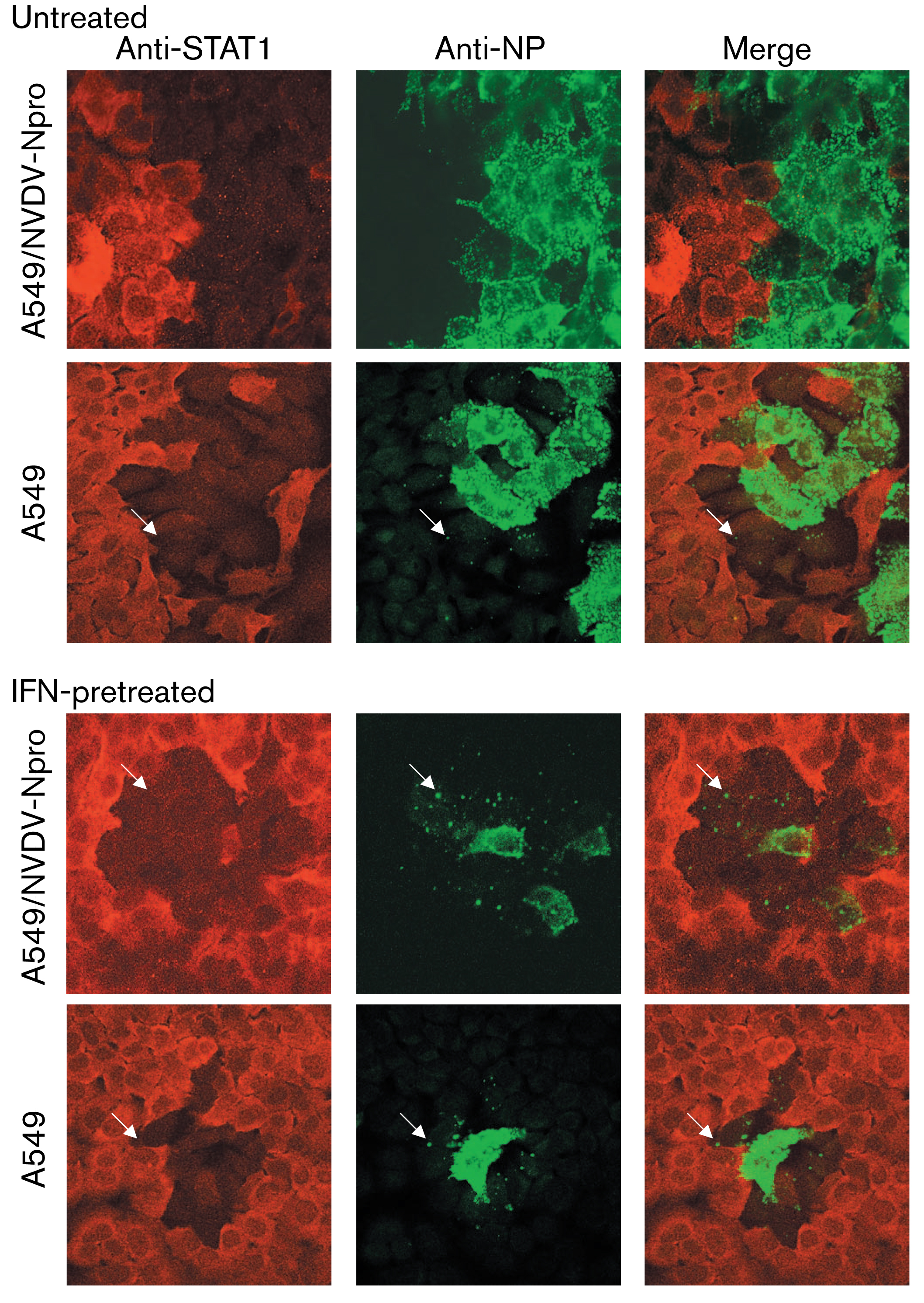

Supplement: [Supplementary Material] [file supp_90_9_2147__vir012047_Fig5.jpg]
